# Dynamic Changes of the Gut Microbiota and Its Functional Metagenomic Potential during the Development of Non-Small Cell Lung Cancer

**DOI:** 10.3390/ijms25073768

**Published:** 2024-03-28

**Authors:** Cuijiao Feng, Na Li, Guangqi Gao, Qiuwen He, Lai-Yu Kwok, Heping Zhang

**Affiliations:** 1 Inner Mongolia Key Laboratory of Dairy Biotechnology and Engineering, Inner Mongolia Agricultural University, Hohhot 010018, China; 18829285357@163.com (C.F.); 18004899551@163.com (N.L.); guangqigao@163.com (G.G.); heidyww@163.com (Q.H.); kwok_ly@yahoo.com (L.-Y.K.); 2 Key Laboratory of Dairy Products Processing, Ministry of Agriculture and Rural Affairs, Inner Mongolia Agricultural University, Hohhot 010018, China; 3 Key Laboratory of Dairy Biotechnology and Engineering, Ministry of Education, Inner Mongolia Agricultural University, Hohhot 010018, China

**Keywords:** non-small cell lung cancer, gut microbiota, murine model, flow cytometry, disease progression

## Abstract

The gut microbiota plays a significant role in tumor pathogenesis by regulating the host metabolism and immune response, and there are few studies focused on tracking changes in the gut microbiota from the onset of lung cancer. Therefore, the aim of our study is combining preclinical and clinical research to thoroughly analyze the signatures of fecal microbiota in lung cancer, which will be useful for early diagnosis and predicting the therapeutic efficacy of lung cancer. The first part of this study analyzed the fecal metagenomic differences between patients with non-small cell lung cancer and healthy subjects, and the second part of this work constructed a murine lung cancer model to monitor changes in mouse fecal metagenomics and T cell immunology during lung cancer progression. We found that the fecal microbiota was altered in both humans and mice with lung cancer, characterized by a significantly reduced microbial diversity and number of beneficial microbes, with increases in potential pathogens. The fecal level of *Akkermansia muciniphila* and the gut metabolic module of the secondary bile acid metabolism were diminished in both humans and mice with lung cancer compared with healthy subjects. Splenomegaly was observed in the lung cancer mice. Flow cytometer analysis of the splenocytes revealed substantial alterations in the proportions of T cell subsets in the lung cancer mice, characterized by significant increases in CD4^+^Foxp3^+^CD25^+^ T regulatory cells (*p* < 0.05) while significant decreases in CD3^+^ T cells (*p* < 0.001), CD4^+^ T cells (*p* < 0.001), and the CD4^+^/CD8^+^ ratio (*p* < 0.01). Vertical and longitudinal analyses of the fecal microbiota of the two mouse groups identified some lung cancer biomarkers (including *Acutalibacter timonensis*, *Lachnospiraceae* bacterium NSJ-38 sp014337195, etc.). The fecal microbiota of the lung cancer mice had a reduced metagenomic potential for neurotransmitters (melatonin, γ-aminobutyric acid, and histamine) compared with healthy mice. In summary, this study found that the diversity, structure, and composition of gut microbiota vary between cancer and healthy conditions, ultimately leading to changes in the potential for functional metagenomics.

## 1. Introduction

Cancer is a major public health issue worldwide. Lung cancer is considered the most prevalent cancer due to its high incidence rate and poor prognosis, and it is also the main cause of cancer-related deaths [1]. A survey reported that, in 2022, there were approximately 2.48 million new cases of lung cancer worldwide, with approximately 1.81 million deaths, ranking first in the world in terms of mortality rate (https://www.uicc.org/news/globocan-2022-latest-global-cancer-data-shows-rising-incidence-and-stark-inequities, accessed on 15 January 2024). Non-small cell lung cancer (NSCLC) accounts for nearly 85% of all lung cancer cases and is the most common pathological type of lung cancer. It is mainly caused by environmental factors and host genetics [2,3]. Smoking is considered a known pathogenic factor for cancer, but statistical data show that less than 15% of NSCLC patients have a smoking history [4]. Although major improvements have been made in the diagnosis and treatment of lung cancer in recent years due to continuous advancement and innovation in medical techniques, patients still do not achieve the best prognosis.

The gut microbiota is linked to human health, and it helps to keep a stable microecological balance. Under healthy conditions, the gut microbiota maintains a beneficial symbiotic relationship with the host through metabolic-immune-neural networks [5]. However, external factors, such as diet, the environment, antibiotic administration, and diseases, can all disrupt the gut microbiota of an individual, leading to gut microbiota imbalance and raising the individual’s cancer susceptibility due to compromises in the gut metabolic capacity and dysregulation of the host immune function [6,7,8]. About 20% of human malignant tumors are associated with microorganisms [9]. The gut microbiota and its metabolites could promote or suppress cancer by regulating host immune and inflammatory responses, profoundly affecting the effectiveness of subsequent radiotherapy, chemotherapy, targeted therapy, and immunotherapy [10,11,12]. Numerous clinical and preclinical studies have reported substantial differences between the gut microbiota of patients with NSCLC and healthy individuals [13,14,15]. Owing to the pathological characteristics of NSCLC, most patients are diagnosed only in the late stage of the disease, with a poor prognosis. Therefore, it would be of interest to search for specific disease-associated biomarkers to allow for early diagnosis and treatment of NSCLC and thus improve patients’ survival and wellness [16].

The gut microbiota is not only closely related to the occurrence of tumors, but can also directly affect the development and metastasis of tumors [17]. For example, it has been observed that, at different stages of gastric cancer development, the gut microbiota structure also undergoes corresponding changes, and different bacterial genera and metabolic pathways will differentiate and enrich at different stages of gastric cancer development [18]. Currently, collecting tissue samples from clinical patients at various disease stages for intergroup comparison is the main method used in research to examine the differences in the gut microbiota at various disease stages. However, this analysis technique disregards individual human differences. Yet, in clinical practice, it is nearly impossible to monitor the dynamic changes in gut microbiota from the occurrence to the development of tumors for the same individual, as it is almost impossible to predict diseases. The gold standard for preclinical research up until this point has been animal testing. Since the evolutionary history of mice and their genes share a high degree of homology with those of humans, tracking disease-associated pathological changes in mouse lung cancer models is feasible, which would provide interesting reference information for human disease.

In this work, nine normal elderly individuals and 12 patients with stage IIIB or IV NSCLC were recruited for the identification of disease-specific signatures in NSCLC. Then, a lung cancer mouse model was constructed by injecting Lewis lung carcinoma (LLC) cells subcutaneously. Lung cancer-associated changes in the gut microbiota were tracked by analyzing changes in the fecal microbiota after different durations of tumor transplant. Meanwhile, flow cytometry analysis was implemented to monitor the changes in the T cell population in the lungs of the cancer model mice throughout the disease’s onset and progression.

## 2. Results

### 2.1. Fecal Microbiota Characteristics of Patients with NSCLC and Healthy Individuals

**Figure 1:**
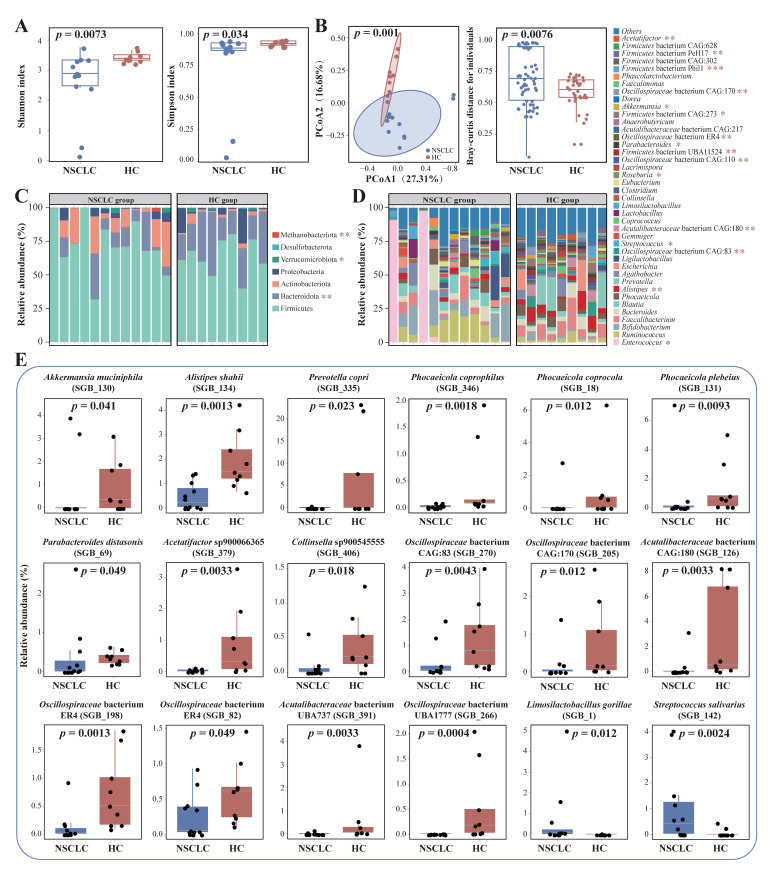
Metagenomics characterization of the gut microbiome of patients with non-small cell lung cancer (NSCLC) and healthy controls (HC). Comparison between the fecal microbiota of the two groups of subjects, NSCLC (n =12) and HC (n = 9) by: (**A**) alpha diversity analysis (represented by the Shannon and Simpson’s diversity indexes); (**B**) principal coordinates analysis (PCoA; Bray–Curtis distance) and intragroup Bray–Curtis distance analysis; and (**C**,**D**) profiling the phylum- and genus-level fecal microbiota composition. For the genus-level data, only major genera comprising over 0.5% of relative abundance in the fecal microbiota dataset are indicated. Significant differentially abundant phyla and genera between the NSCLC and HC groups are indicated by single, double, and triple asterisk(s), representing *p* < 0.05, *p* < 0.01, and *p* < 0.001, respectively, Wilcoxon test. Blue asterisk(s) mean(s) that the taxon is more abundant in the NSCLC group than the HC group, and vice versa for red asterisk(s). (**E**) Significantly different species-level genomic bins (SGBs) identified between the NSCLC and HC groups are shown. The *p*-value of each pairwise comparison by the Wilcoxon test is shown.

The α diversity (Shannon and Simpson’s diversity indexes) of the fecal microbiota of subjects with or without NSCLC was assessed. The results showed that the fecal microbiota of patients with NSCLC had a significantly lower α diversity than those of healthy subjects (*p* = 0.0073 and *p* = 0.034 for Shannon and Simpson’s diversity indexes, respectively; Figure 1A).

Then, the β diversity of the fecal microbiota of the two groups of subjects was assessed by PCoA (Bray–Curtis distance). On the PCoA score plot, the symbols representing the fecal samples of the two groups exhibited a group-based clustering pattern (*p* = 0.001, Figure 1B), suggesting that a significant difference exists between the fecal microbiota of the subject groups. In addition, the fecal microbiota of subjects with NSCLC had a significantly higher level of intragroup Bray–Curtis distance than those of healthy individuals (*p* = 0.0076, Figure 1B). These results suggested that the fecal microbiota diversity and structure were significantly different between the subjects with and without NSCLC.

We further analyzed the composition of the phylum- and genus-level fecal microbiota of the two groups of subjects (Figure 1C,D). The fecal microbiota of all subjects mainly belonged to seven phyla, and the top three phyla were Firmicutes, Bacteroidota, and Actinobacteriota, accounting for 95.86% and 92.92% of the total bacterial sequences in the NSCLC and HC groups, respectively. Some interesting differences were noted. At the phylum level (Figure 1C), the fecal microbiota of NSCLC subjects had significantly more Methanobacteriota (*p* = 0.004) but fewer Bacteroidota and Verrucomicrobiota compared with those of the healthy subjects (*p* = 0.001 and *p* = 0.015, respectively). At the genus level (Figure 1D), there were 16 significantly differently abundant genera between the two groups of subjects. Significantly more *Streptococcus* (*p* = 0.028) and *Enterococcus* (*p* = 0.049) were found in the fecal microbiota of subjects with NSCLC than those of the healthy subjects, while *Acetatifactor* (*p* = 0.006), *Firmicutes* bacterium PeH17 (*p* = 0.003), *Firmicutes* bacterium Phil1 (*p* = 0.001), *Oscillospiraceae* bacterium CAG:170 (*p* = 0.002), *Akkermansia* (*p* = 0.041), *Firmicutes* bacterium CAG:273 (*p* = 0.015), *Oscillospiraceae* bacterium ER4 (*p* = 0.004), *Parabacteroides* (*p* = 0.023), *Firmicutes* bacterium UBA11524 (*p* = 0.002), *Oscillospiraceae* bacterium CAG:110 (*p* = 0.003), *Roseburia* (*p* = 0.049), *Acutalibacteraceae* bacterium CAG:180 (*p* = 0.003), *Oscillospiraceae* bacterium CAG:83 (*p* = 0.003), and *Alistipes* (*p* = 0.004) had an opposite trend in relative abundance.

At the SGB level, 18 significantly differently abundant SGBs were identified between the two groups (Figure 1E). Significantly more *Limosilactobacillus gorillae* (SGB_1, *p* = 0.012) and *Streptococcus salivarius* (SGB_142, *p* = 0.002) were found in the fecal microbiota of subjects with NSCLC compared with those of the healthy subjects, while the other 16 SGBs showed an opposite trend, including *Akkermansia muciniphila* (*A. muciniphila*; SGB_142, *p* = 0.002), *Alistipes shahii* (SGB_134, *p* = 0.001), *Prevotella copri* (SGB_335, *p* = 0.023), and *Phocaeicola coprophilus* (SGB_346, *p* = 0.002), *Phocaeicola coprocola* (SGB_18, *p* = 0.012), *Phocaeicola plebeius* (SGB_131, *p* = 0.009), *Parabacteroides distasonis* (SGB_69, *p* = 0.049), *Acetatifactor* sp900066365 (SGB_379, *p* = 0.003), *Collinsella* sp900545555 (SGB_406, *p* = 0.018), *Oscillospiraceae* bacterium CAG:83 (SGB_270, *p* = 0.004), *Oscillospiraceae* bacterium CAG:170 (SGB_205, *p* = 0.012), *Acutalibacteraceae* bacterium CAG:180 (SGB_126, *p* = 0.003), *Oscillospiraceae* bacterium ER4 (SGB_198, *p* = 0.001), *Oscillospiraceae* bacterium ER4 (SGB_82, *p* = 0.049), *Acutalibacteraceae* bacterium UBA737 (SGB_391, *p* = 0.003), and *Oscillospiraceae* bacterium UBA1777 (SGB_266, *p* = 0.001). These results, together, suggested that the gut microbiota of subjects with and without NSCLC differed substantially in terms of composition.

To investigate the differences in the functional genomic potentials of the fecal microbiota between the two subject groups, we further compared the fecal microbiota data subsets of the two groups, revealing a total of 23 significantly differently abundant GMMs between the two subject groups (Table 1). All of them had a significantly lower abundance in the fecal microbiota of patients with NSCLC compared with those of the healthy subjects, and they were mainly GMMs that are involved in the synthesis/degradation of short-chain fatty acids (SCFAs, including butyrate, isovalerate, acetate, propionate), an amino acid (tryptophan), neurotransmitters (melatonin, γ-aminobutyric acid [GABA], and histamine), and other bioactive substances (secondary bile acids, quinolinic acid, polyunsaturated fatty acid [PUFAs], and menaquinone).

### 2.2. Lung Tumor Transplantation

**Figure 2:**
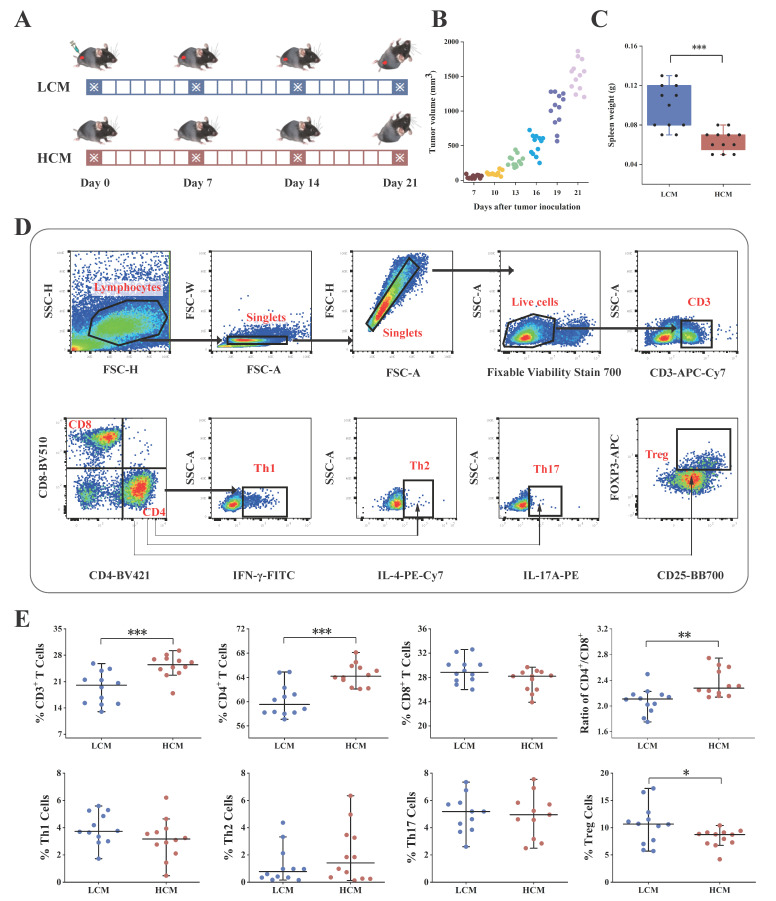
Construction of lung cancer mice (LCM) and immune characterization. (**A**) Experimental design. Two groups of mice (n = 12 per group) were included in this experiment, including lung cancer mice (LCM) and healthy control mice (HCM). Tumor transplantation (day 0) was achieved by subcutaneously injecting LLC1 cells into the LCM, while the HCM received no treatment. Fecal samples were collected on Days 0, 7, 14, and 21 after tumor transplantation. Mice were sacrificed for spleen dissection for flow cytometry analysis. (**B**) Tumor growth. (**C**) Spleen weight of the two mouse groups. (**D**) Flow gate strategy. (**E**) Composition of major T lymphocyte subpopulations in the spleens of the two mouse groups. * *p* < 0.05, ** *p* < 0.01 and *** *p* < 0.001, respectively; Wilcoxon test.

A lung cancer mouse model was constructed by injecting LLC1 cells subcutaneously (Figure 2A). After one week, mice in the LCM group began to develop palpable tumor tissue at the right flank (mean tumor volume = 50.82 mm^3^ ± 6.89). On day 10, the average tumor volume grew by 99.65 mm^3^ ± 9.29. However, on day 13, the tumor volume started to increase exponentially, reaching 280.49 mm^3^ ± 23.25, until day 21 when the mice were euthanized (Figure 2B).

### 2.3. T Cell Immunology in Lung Cancer and Healthy Mice

On day 21, the mouse spleens were collected. The weights of the spleens in the LCM group were significantly higher than those of the healthy mice without LLC1 cell inoculation (*p* < 0.001; Figure 2C), suggesting a strong immunological activity against the tumors in tumor-bearing mice.

Then, T lymphocytes were isolated from the spleens of the two groups of mice for flow cytometry analysis. The flow gate strategy used in this study is shown in Figure 2D. The mice in the LCM group had significantly lower levels of CD3^+^ T cells and CD4^+^ T cells, and a lower CD4^+^/CD8^+^ ratio, than the healthy mice (*p* < 0.001, 0.001, and 0.01, respectively; Figure 2E). Moreover, the mice in the LCM group had a significantly higher proportion of CD4^+^Foxp3^+^CD25^+^ Treg cells than the healthy mice (*p* < 0.05; Figure 2E). The altered distribution of some T cell subsets in the tumor-bearing mice compared with the healthy ones might suggest immunity disruption in the lung cancer mice.

### 2.4. Fecal Microbiota in Mice with and without Lung Cancer

**Figure 3:**
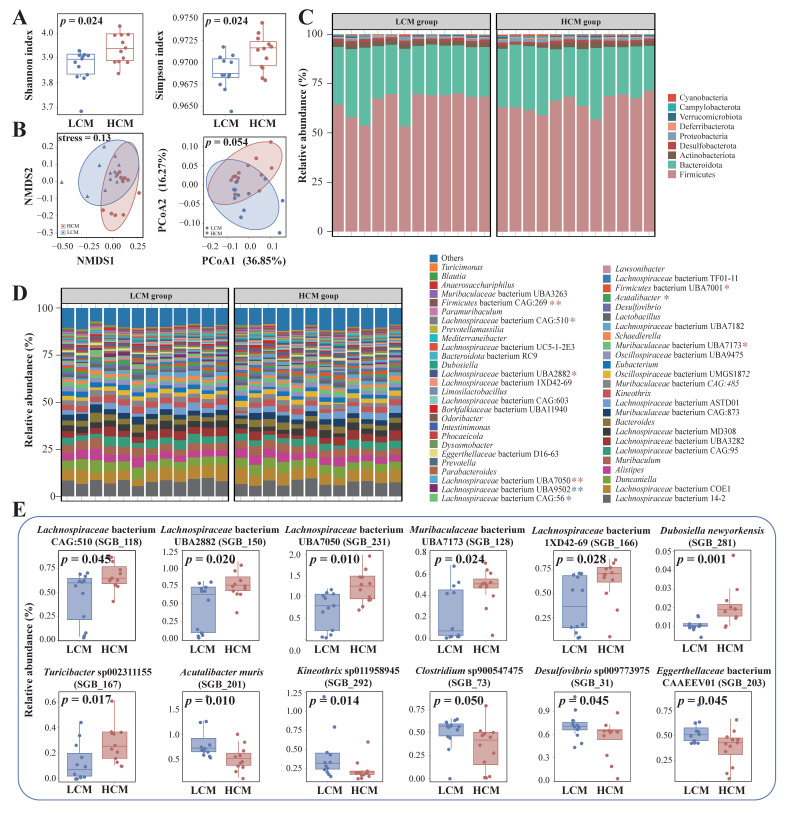
Metagenomics characterization of the gut microbiome of lung cancer mice and wild-type mice. (**A**) Differences in alpha diversity between the LCM and HCM groups based on the Shannon and Simpson indexes. LCM, lung cancer mice group; HCM, healthy control mice group. (**B**) Beta diversity differences between the NSCLC group and HC group were estimated by non-metric multidimensional scaling (NMDS) and principal coordinates analysis (PCoA). (**C**) Bacteria composition at phylum and (**D**) genus levels between lung cancer mice and wild-type mice. The displayed phylum levels are all phyla and the dominant genus was screened with the average relative abundance greater than 5%. The different phyla and genera between NSCLC and HC groups are marked with an asterisk, and the blue and red asterisks, respectively, represent that the bacterium has a high relative abundance in the LCM group and HCM group. (**E**) Differently changed SGBs between LCM and HCM groups. LCM and HC groups both include 12 mice. * *p* < 0.05, and ** *p* < 0.01, respectively; Wilcoxon test.

The fecal microbiota of the LCM and healthy mice were analyzed in depth. Firstly, the α diversity of their fecal microbiota was comparatively analyzed (Figure 3A). The fecal microbiota of mice in the LCM group had lower values of Shannon and Simpson’s diversity indexes compared with those of the healthy mice (*p* = 0.024 in both cases), which was consistent with the results of the human subjects. The β-diversity of the fecal microbiota dataset was visualized with both non-metric multidimensional scaling (stress value = 0.13) and PCoA (Bray–Curtis distance). The symbols representing the two groups of mice form group-based clustering patterns in the score plots of both analyses, although some overlapping was observed (Figure 3B), suggesting the existence of mild differences between the fecal microbiota structures of the two groups of mice.

Then, we analyzed the phylum- and genus-level fecal microbiota compositions (Figure 3C,D). The mouse fecal microbiota mainly belonged to nine different phyla, and the top three phyla of the two groups of mice were Firmicutes, Bacteroidota, and Actinobacteriota, accounting for 96.14% and 95.43% of the LCM and HCM groups, respectively (Figure 3C). No significant difference was observed in any of the nine phyla. At the genus level, a total of 52 major genera (those ≥0.5% of the total sequences) were identified (Figure 3D). Comparative abundance analysis found that nine genera showed significant differential abundances between the two groups. The LCM group had more *Lachnospiraceae* bacterium UBA9502 ((*p* = 0.01), *Lachnospiraceae* bacterium CAG:56 (*p* = 0.02), and *Acutalibacter* ((*p* = 0.045) than the HCM group, while six genera showed an opposite trend, including *Firmicutes* bacterium CAG:269 (*p* = 0.002), *Lachnospiraceae* bacterium CAG:510 (*p* = 0.045), *Lachnospiraceae* bacterium UBA2882 (*p* = 0.02), *Lachnospiraceae* bacterium UBA7050 (*p* = 0.01), *Firmicutes* bacterium UBA7001 (*p* = 0.014), and *Muribaculaceae* bacterium UBA7173 (*p* = 0.045).

At the SGB level (Figure 3E), seven SGBs were significantly more abundant in the fecal microbiota of the HCM group than in those of the LCM group, including *Lachnospiraceae* bacterium CAG:510 (*p* = 0.045), *Lachnospiraceae* bacterium UBA2882 (*p* = 0.02), *Lachnospiraceae* bacterium UBA7050 (*p* = 0.01), *Muribaculaceae* bacterium UBA7173 (*p* = 0.045), *Lachnospiraceae* bacterium 1XD42-69 (*p* = 0.028), *Dubosiella newyorkensis* (*p* = 0.001), and *Turicibacter* sp002311155 (*p* = 0.017). Five SGBs were significantly more abundant in the fecal microbiota of the LCM group compared with those of the HCM group, including *Acutalibacter muris* (*p* = 0.010), *Kineothrix* sp011958945 (*p* = 0.050), *Clostridium* sp900547475 (*p* = 0.045), *Desulfovibrio* sp009773975 (*p* = 0.045), and *Eggerthellaceae* bacterium CAAEEV01 (*p* = 0.045); these SGBs might have a greater competitive advantage in the gut of mice with lung cancer.

Functional annotation of the fecal SGBs identified a total of 19 GMMs. Only one differentially abundant SGB was identified, namely secondary bile acid biosynthesis (GMM014), which was significantly deprived in the LCM group compared with the HCM group (*p* = 0.028; Figure S1).

### 2.5. Fecal Microbiota at Different Tumor Progression Stages

To explore the dynamic changes in the host gut microbiota in the onset and development of lung cancer, we collected fecal samples from the lung cancer and healthy mice at different time points of tumor transplant (days 1, 7, 14, and 21, corresponding to HCM_ T1, HCM_T2, HCM_T3, and HCM_T4 for the healthy control group, and LCM_T1, LCM_T2, LCM_T3, and LCM_T4 for the lung cancer group, respectively).

**Figure 4:**
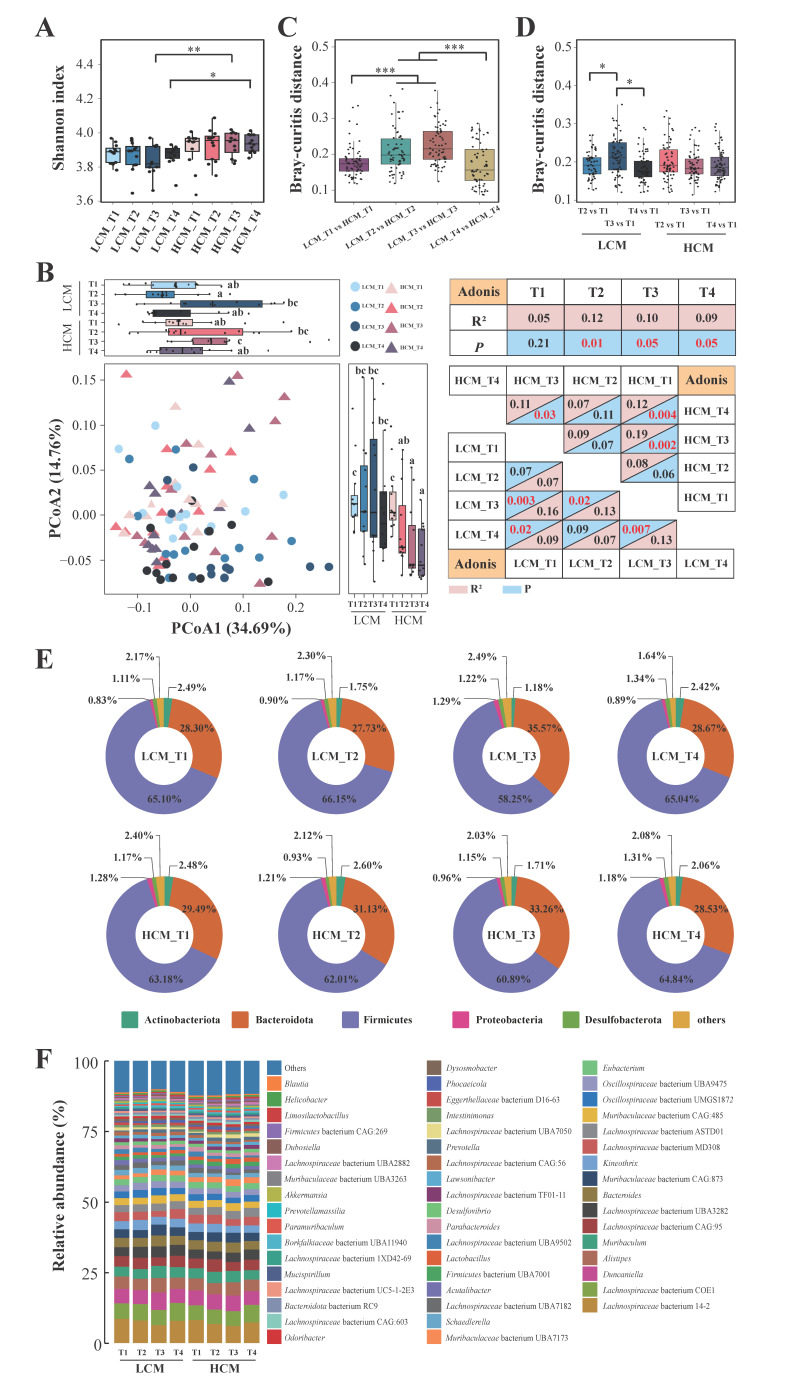
Dynamic changes in the gut microbiome during lung cancer development. (**A**,**B**) Shannon index and principal coordinates analysis (PCoA) of the fecal microbiota of lung cancer mice (LCM) and healthy control mice (HCM) at the four sampling time points (T1, T2, T3, and T4, representing days 0, 7, 14, and 21). The intersubgroup differences in PCoA1 and PCoA2 were evaluated by Wilcoxon tests, and significant differences between subgroups are indicated by different letters with a cut-off confidence level of 95%. The intersubgroup differences in the Bray–Curtis distance between (**C**) LCM and HCM groups at the same time point and (**D**) different time points of the same group were compared. (**E**) The donut diagrams and (**F**) the stacked bar chart illustrate the composition of the top five dominant phyla and the genus-level bacterial fecal microbiota composition in each subgroup. * *p* < 0.05, ** *p* < 0.01, and *** *p* < 0.001, respectively; Wilcoxon test.

We first assessed the changes in the α diversity of the fecal microbiota of the two groups of mice (Figure 4A). The fecal microbiota of the HCM group had an overall higher level of Shannon diversity compared with those of the LCM group, though significant differences were only observed at the later time points, i.e., days 14 and 21 of tumor transplant (*p* < 0.01 and 0.05, respectively; Figure 4A). The Shannon diversity did not show a significant trend of change as the lung cancer progressed (Figure 4A).

Then PCoA (Bray-Curtis distance) and Adonis tests were performed to investigate the β diversity analysis of the fecal microbiota of the two groups of mice (Figure 4B). On the PCoA score plot, no clear clustering pattern could be identified; however, the symbols representing the fecal microbiota of early time points for the LCM (LCM_T1, LCM_T2) were located in the upper half of the score plot, while those representing the later time points (LCM_T3, LCM_T4) were mainly aggregated at the lower part of the score plot (Figure 4B), suggesting a gradual change in the fecal microbiota structure with the time of tumor transplantation. Such a trend was not as obvious in the HCM group. The fecal microbiota of the control group showed significant changes from day 14 compared with baseline, and the changes persisted until the end of the experiment (HCM_T1 group versus HCM_T3 group, *p* = 0.002, R^2^ = 0.19; HCM_T1 group versus HCM_T4 group, *p* = 0.004, R^2^ = 0.12; HCM_T3 group versus HCM_T4 group, *p* = 0.03, R^2^ = 0.11; Figure 4B). As the lung cancer progressed, the structure of the fecal microbiota of the LCM group also showed significant differences compared with that at the baseline (LCM_T1 versus LCM_T3, *p* = 0.003, R = 0.16; LCM_T1 versus LCM_T4, *p* = 0.03 and R^2^ = 0.09; Figure 4B). However, a significant difference was also noted between days 7 and 14 of the LCM group (LCM_T2 vs. LCM_T3, *p* = 0.02, R^2^ = 0.13; Figure 4B), suggesting an earlier fecal microbiota structure change in the LCM group compared to the HCM group. The results of Adonis tests and Bray–Curtis distance analysis also revealed significant intergroup differences between the fecal microbiota of the two groups at days 7, 14, and 21 (*p* = 0.01, 0.05, 0.05, respectively; Figure 4B,C). Moreover, the intragroup Bray–Curtis distance of the LCM group but not the HCM group also showed significant differences (Figure 4D). These results suggested that the structure of the fecal microbiota of both groups underwent substantial changes during the mouse experiment, but the magnitude of changes was greater in the LCM group compared with the HCM group.

To pinpoint the exact variation in the fecal microbiota over the lung cancer progression, we characterized the fecal microbiota composition at different taxonomic levels (Figure 4E). At the phylum level, the top three phyla at all four time points in both groups were Firmicutes, Bacteroidota, and Actinobacteriota, except for the fecal microbiota of LCM_T3, in which Proteobacteria was the third top phylum instead of Actinobacteriota (Figure 4E). At the genus level, the profile of the dominant genera did not change drastically during the entire period (Figure 4F). The top three genera were consistently *Duncaniella*, *Lachnospiraceae* bacterium COE1, and *Lachnospiraceae* bacterium 14-2.

### 2.6. SGB-Level Disease-Driven Gut Microbiota Responses

**Figure 5:**
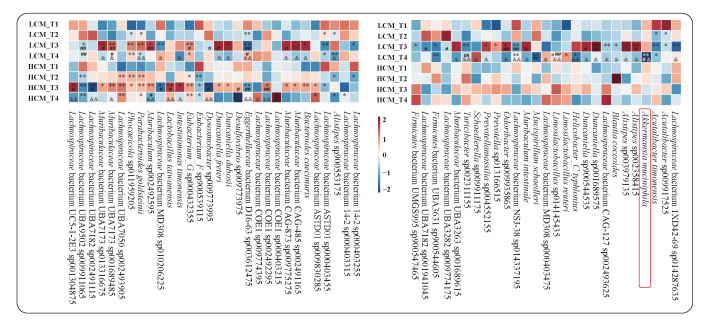
Significantly altered fecal microbiota during lung cancer development. Heatmaps showing the differentially changed species in the healthy control mice (HCM; left panel) and lung cancer mice (LCM; right panel) at different time points of tumor transplantation (T1, T2, T3, and T4, representing days 0, 7, 14, and 21). Only major species with an average relative abundance greater than 5% are included. The color scale represents the microbial relative abundance; the intensity of red and blue indicates more and less abundant, respectively. Significant differences between sample pairs were evaluated by Wilcoxon tests; * represents T1 versus T2, T3, and T4; # represents T2 versus T3, T2 versus T4, T3 versus T4; & represents T3 versus T4. Single and double symbols represent *p* < 0.05, *p* < 0.01, and *p* < 0.001, respectively.

We then delved deeper into the responses of disease-associated fecal microorganisms at the SGB level. Firstly, we identified the spectrum of SGBs that would naturally fluctuate with the age of healthy mice (inclusing only SGBs of relative abundance ≥ 0.5%, Figure 5, left panel). In total, 30 species were identified, 19 of which were also found to vary in relative abundance in the LCM group. The variations in these SGBs were considered to be related to natural changes in the age and intestinal physiology of the mice. Specifically, compared with HCM_T1, *Lachnospiraceae* bacterium UBA7050 sp002493905, *Parabacteroides goldsteinii*, and *Eubacterium_G* sp000432355 in HCM_T2 (*p* = 0.004, *p* = 0.002, *p* = 0.020), HCM_T3 (*p* = 0.001, *p* = 0.003, *p* = 0.001) and HCM_T4 (*p* = 0.001, *p* = 0.014, *p* = 0.008) significantly increased, while *Lachnospiraceae* bacterium UBA9502 sp009911065 and *Eubacterium_F* sp900539115 were significantly reduced in three time points of the HCM group (*p* = 0.004 and *p* = 0.007 in HCM_T2 group; *p* = 0.001 and *p* = 0.012 in HCM_T3 group; *p* = 0.001 and *p* = 0.017 in HCM_T4 group). In addition, the *Phocaeicola* sp011959205 in HCM_T2 (*p* = 0.007) and HCM_T3 (*p* = 0.017) was significantly increased, and the *Lachnospiraceae* bacterium ASTD01 sp009830285 in HCM_T3 (*p* = 0.028) and HCM_T4 (*p* = 0.014) was significantly increased, while *Alistipes* sp900553175 showed a significant decrease in HCM_T2 (*p* = 0.020) and HCM_T4 (*p* = 0.017). The *Lachnospiraceae* bacterium 14-2 sp000403255 in the HCM_T2 was specifically higher than in the HCM_T1 (*p* = 0.017). The remaining 21 species only began to change on day 14. For example, compared to Day 7, the relative abundance of *Lactobacillus taiwanensis* significantly increased on day 14 (*p* = 0.017) and day 21 (*p* = 0.001).

We then searched for differential SGBs between the HCM and LCM groups that were specific for the lung cancer after excluding the naturally changed ones, identifying a total of 27 (Figure 5, right panel). These were SGBs that are responsive to different stages of tumor progression. For example, *Acutalibacter* sp009917525 and *Acutalibacter timonensis* changed during early tumor growth at day 7 (*p* = 0.014 and *p* = 0.014, respectively). In the mid-to-late stage, more species exhibited significant abundance changes. Notably, significantly fewer *A. muciniphila* were found in LCM_T4 compared with HCM_T1, HCM_T2, and HCM_T3 at day 21 (*p* = 0.003, *p* = 0.017, and *p* = 0.012, respectively). Consistently, *A. muciniphila* was also identified as a lung cancer-responsive species in human fecal samples of subjects with lung cancer.

### 2.7. Changes in the Fecal Microbiota-Encoded Metabolic Modules in Lung Cancer Mice

**Figure 6:**
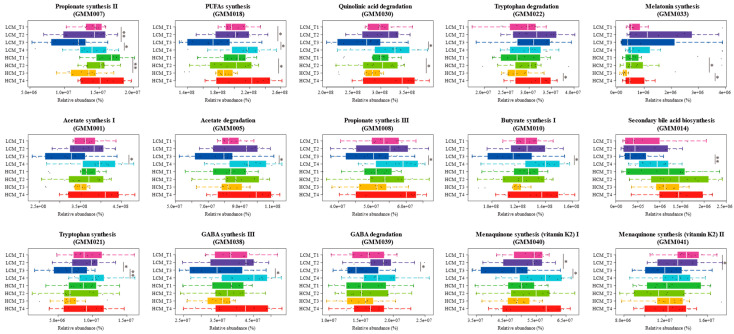
Significantly changed gut metabolic modules (GMMs) during lung cancer development in mice. Microbiota changes at different time points (T1, T2, T3, and T4, representing days 0, 7, 14, and 21 after tumor transplantation) were monitored. Significant differences were evaluated with Wilcoxon tests; * *p* < 0.05, and ** *p* < 0.01, respectively. LCM = lung cancer mice; HCM = healthy control mice; GABA = γ-aminobutyric acid; PUFA = polyunsaturated fatty acid.

We then analyzed the GMMs encoded by gut microbiota to reveal their functional changes at different stages of lung cancer development. A total of 15 differential GMMs were identified (Figure 6). The GMMs of propionate synthesis II, polyunsaturated fatty acid synthesis, and quinolinic acid degradation decreased significantly in HCM_T3 (*p* = 0.008, *p* = 0.043, *p* = 0.049 in HCM_T1 vs. HCM_T3, respectively), while there was a significant increase in LCM_T4 (*p* = 0.049, *p* = 0.033, *p* = 0.033 in LCM_T3 vs. LCM_T4, respectively). In addition, the GMMs of tryptophan degradation and melatonin synthesis were specifically enriched in the HCM group (*p* = 0.024, *p* = 0.033 in HCM_T3 vs. HCM_T4, respectively). The remaining ten GMMs showed changes within the LCM group: significant increases in acetate synthesis I, acetate degradation, propionate synthesis III, butyrate synthesis I, secondary bile acid biosynthesis, and GABA synthesis III (*p* = 0.033, *p* = 0.038, *p* = 0.018, *p* = 0.033, *p* = 0.009, *p* = 0.038 in LCM_T3 vs. LCM_T4, respectively); significant decreases in GABA degradation and menaquinone synthesis (vitamin K2) II (*p* = 0.049 in LCM_T2 vs. LCM_T3; *p* = 0.033 in LCM_T1 vs. LCM_T3). The ones that increased first and then decreased included: tryptophan synthesis (*p* = 0.038 in LCM_T2 vs. LCM_T3; *p* = 0.007 in LCM_T3 vs. LCM_T4) and menaquinone synthesis (vitamin K2) I (*p* = 0.043 in LCM_T1 vs. LCM_T3; *p* = 0.015 in LCM_T3 vs. LCM_T4). These GMMs might play an essential role in the progression of lung cancer.

## 3. Discussion

The gut microbiota and its metabolites play a critical role in the host’s health. They can interfere with the host’s regular physiological functions and impact the body’s immune system, increasing their susceptibility to malignant tumors and influencing the subsequent clinical treatment strategy and therapeutic effects. Therefore, this study used animal and clinical studies to examine the gut microbiota characteristics in lung cancer subjects and tracked the dynamic changes in gut microbiota at various stages of disease development using a murine lung cancer model. To our knowledge, this study is the first to examine the gut microbiota characteristics in lung cancer from both preclinical and clinical experimental perspectives.

We first compared the gut microbiota of patients with NSCLC to those of healthy people and then validated our results in an experimental mouse lung cancer model. Both parts of this work found that the gut microbiota was altered in subjects with lung cancer, characterized by diminished microbial diversity, obvious structural changes, and alterations in the abundance of certain disease-associated biomarker bacterial species. Our results are in line with the results of previous works [13,19]. Therefore, identifying alterations in the gut microbiota in people at high risk for lung cancer could be a useful tool for early clinical diagnosis and treatment of the disease.

Owing to reduced activity and inadequate food and water intake, cancer patients are often prone to constipation [20]. Our study found that lung cancer patients had a relatively high amount of intestinal *Methanobacteriota*, which mainly contains some methane-producing bacteria. These bacteria can produce methane through the anaerobic fermentation of sugars, thereby inhibiting intestinal peristalsis and worsening constipation [21]. Significantly more *Enterococcus* was found in the fecal samples of patients with NSCLC. *Enterococcus* is a common human gut resident, but some members of this genus have been reported to promote the binding of nitrite and amines in food to form a highly carcinogenic nitrosamine [22]. Meanwhile, the abundance of two lactic acid bacterial species, *Streptococcus salivarius* and *Limosilactobacillus gorillae*, was higher in the fecal samples of patients with NSCLC than in healthy individuals, but whether these microbes played any role in tumor occurrence and development requires further investigation. Notably, the fecal microbiota of patients with NSCLC had diminished levels of some beneficial bacteria, such as *A. muciniphila*, *Oscillospiraceae* bacteria, and *Alistipes shahii*. Some *Oscillospiraceae* are known to produce SCFAs [23], while *A. muciniphila* and *Alistipes shahii* are found to associate with the responsiveness and effectiveness of cancer immunotherapy [24,25]. Obvious differences in the predicted functional potential of the gut microbiota between the two groups of patients were also noted: the fecal microbiota of patients with NSCLC had fewer GMMs which encode pathways relating to the metabolism and biosynthesis of SCFAs, amino acids, and secondary bile acids compared with those of healthy individuals.

We have also identified several interesting mouse lung cancer gut bacterial biomarkers, which were enriched in the fecal microbiota of mice with lung cancer, including *Acutalibacter muris*, *Clostridium* sp900547475, *Desulfovibrio*, and *Kineothrix. Acutalibacter muris* has been reported as a biomarker of colorectal cancer [26], while *Clostridium* sp900547475 could be used to predict the treatment sensitivity [27]. In addition, members of the *Desulfovibrio* genus are considered pathogenic gut bacteria, as a high abundance of *Desulfovibrio* could promote gastrointestinal inflammation, promoting lung cancer development [28,29]. *Kineothrix* is associated with depression [30]. Cancer has a serious psychological impact on patients, causing excessive stress, long-term anxiety, and even depression. The gut microbiota can be regarded as the ‘ventral brain’ that influences the host’s psychological state through the brain–gut axis [31,32,33]. Thus, it is not surprising to see enrichment in some depression-associated bacteria in the fecal microbiota of lung cancer mice compared with healthy mice. In line with our results of comparison between the fecal microbiota of patients with and without lung cancer, some beneficial bacteria (mainly *Lachnospiraceae*) had a diminished abundance in the fecal microbiota. Members of the *Lachnospiraceae* family are known SCFA-producers [34]. On the other hand, the functional potentials encoded by the fecal microbiota of lung cancer mice and healthy control mice were highly similar, except that the gene abundance of the secondary bile acid synthesis module was significantly lower in LCM than in healthy mice.

The phylogenetic composition of the gut microbiota varies between mice and humans due to intrinsic differences in the physiological structure and biochemical environment of the gut. [35]. Another reason for this difference is that rodents, like mice, reintroduce the gut microbiota into the small intestine through self-feeding feces (i.e., self-reinoculation), changing the gut’s internal environment and microbial community in the process [36]. Despite the above-mentioned inevitable drawbacks, it is challenging to meet research needs solely based on samples from humans due to ethical and safety concerns when it comes to developing disease models to understand causal relationships. Mice are commonly used as models in biological experiments because they are simple to care for and have short gestation periods, making them valuable resources and tools for scientific study. Even though the disease-associated microbial biomarkers in lung cancer mice and NSCLC patients varied, the GMM of the secondary bile acid synthesis pathway was conspicuously the metabolic pathway that was differentially abundant between humans/mice with and without lung cancer.

Another advantage of experimentation with animal disease models is that it allows for a wider range of horizontal studies. For example, it is possible to track the dynamic changes of certain indicators over a long period after constructing the disease model. The gut microbiota is closely associated with health and disease, and the composition and structure of the intestinal microbiota varies with the host’s health conditions and changes, particularly in severe diseases, including cancer. Thus, by constructing an LCM model, it is possible to associate the changes in the mouse gut microbiota and T cell immunity after tumor transplantation with the disease progression. Thus, in the second part of this work, we tracked the changes in the gut microbiota throughout lung cancer development using a murine model. Obvious changes were observed in members of the *Lachnospiraceae* family. Although the *Lachnospiraceae* family is generally considered a core beneficial group of bacteria in the gut due to their role as major SCFA producers, various *Lachnospiraceae* taxa are linked to a variety of intra- and extra-intestinal diseases [37]. This study found that the relative abundance of certain species of *Lachnospiraceae* decreased around day 21 of cancer progression (including *Lachnospiraceae* bacterium 14-2 sp000403255, *Lachnospiraceae* bacterium ASTD01 sp000403455, *Lachnospiraceae* bacterium UBA7182 sp002491115, *Lachnospiraceae* bacterium UBA9502 sp009911065, *Lachnospiraceae* bacterium MD308 sp010206225, and *Eubacterium_F* sp900539115), while some other species of the same family increased in abundance (including *Lachnospiraceae* bacterium 14-2 sp000403315, *Lachnospiraceae* bacterium ASTD01 sp009830285, *Lachnospiraceae* bacterium COE1 sp000403215, *Lachnospiraceae* bacterium COE1 sp002492295, *Lachnospiraceae* bacterium UBA7050 sp002493905). Moreover, we observed an increased relative abundance in *Lactobacillus taiwanensis*. The functional genomics analysis of these microbes revealed that they mainly participate in the synthesis or degradation of SCFAs, amino acids, and other metabolites.

In the early stages of lung cancer, we observed a significant decrease in the *Acutalibacter* genus. The body’s immune system is compromised during the middle and late stages of tumor development [38], and the microbial ecosystem is undergoing progressive changes, with significant alterations in multiple species. Moreover, intriguingly, the results from both our murine and human studies found that the level of fecal *A. muciniphila* significantly dropped under the disease state (at day 21 in the LCM model). Numerous studies have demonstrated the close connection between *A. muciniphila* and the immune system homeostasis of the host, along with the way it may boost the therapeutic effectiveness of immune checkpoint therapy [39]. These findings suggested that *A. muciniphila* is a beneficial microbe in preventing cancer and enhancing the effect of cancer therapy and that it could be used as a biomarker for cancer diagnosis, predicting drug treatment efficacy, and even the prognosis of the disease. Future research might focus on *A. muciniphila* surveillance. Further functional metagenomics analysis found that the most significant changes in the gut metabolism modules of LCM were related to neurotransmitter synthesis/degradation. A previous study reports that colon and lung cancer cells could secrete GABA, a key inhibitory neurotransmitter found in mammalian central nervous systems that stimulates tumor growth and impairs tumor immunity [40].

The spleen is an important immune organ in the body, and splenomegaly suggests that the body’s immune homeostasis is disrupted. The LCM exhibited severe splenomegaly, which could be regarded as the host immune response against the transplanted lung tumor. We thus used flow cytometry to analyze the different subsets of T lymphocytes in the spleen of the LCM. Microorganisms have a powerful impact on both immunity and tumors and are considered a key factor connecting the two [40]. Some gut bacteria have been shown to promote the proliferation of certain immunosuppressive cells (such as Treg and myeloid-derived suppressor cells), diminishing the intensity of cellular immunity, assisting the immune escape of tumor cells, and promoting the occurrence and development of tumors [41]. For example, typical *Fusobacterium nucleatum* could recruit tumor-infiltrating bone marrow cells, inhibiting T cells and natural killer cells [42]; *Peptostreptococcus anaerobius* could increase tumor-associated macrophages and neutrophils, ultimately leading to tumor progression [43]. On the contrary, there may also be beneficial gut bacteria that help counter tumors by strengthening the host’s immunity. For example, some *Clostridium* strains could increase the amount of natural killer cells in the liver, thereby enhancing the host anti-tumor immunity and inhibiting liver tumor progression [44]. In our study, some species that are known to be involved in host immune response were found to have decreased in the fecal microbiota of LCM compared with the non-cancerous mice, particularly *Blautia coccoides* and *A. muciniphila*. *Blautia* was enriched in the gut microbiota of patients with NSCLC who were responders to immune checkpoint inhibitors cancer immunotherapy compared with non-responders [45]. The lack of *A. muciniphila* might lead to the infiltration of myeloid-derived suppressor cells, triggering inflammation and tumor progression [17]. Although it has been demonstrated in clinical practice that fecal microbiota transplantation may enhance the efficacy of anti-PD-1 therapy in some cancer patients, the effects of the enhancement are inconsistent [46], suggesting that gut microbiota-driven regulation of the effectiveness of tumor immunotherapy is complex, which is worthy of further investigation.

In summary, this study comparatively analyzed the fecal metagenomics differences between cancerous and healthy humans and mice. Splenomegaly was observed in LCM, suggesting the disruption of the host’s immune homeostasis, which may be related to gut dysbiosis. Our fecal metagenomics analysis revealed that lung cancer development was associated with alterations in the gut microbiota diversity, structure, and composition, causing changes in the functional metagenomic potential of pathways related to the metabolism of SCFAs, amino acids, and neurotransmitters. The temporal dynamics analysis of gut microbiota in LCM revealed some interesting stage-specific lung cancer biomarker species (such as *Acutalibacter timonensis*, and *Acutalibacter* sp009917525 in the early stage of lung cancer; *Muribaculaceae* bacterium UBA3263 sp001689615, *Limosilactobacillus reuteri*, and *Schaedlerella* sp009911175 in the late stage of lung cancer). Notably, *A. muciniphila* was a common lung cancer biomarker species found in both humans and mice. The data, however, are not representative and comprehensive because of this study’s small clinical sample size and failure to consider dietary influences. To validate the results of this study, additional research with a larger sample size will be required in the future.

## 4. Materials and Methods

### 4.1. Subjects and Clinical Samples Collection

We collected fresh feces from 9 normal elderly volunteers and 12 patients diagnosed with stage IIIB or IV NSCLC before they started clinical treatment. All participants had provided written informed consent before this study. The age range of the nine normal elderly volunteers is between 63 and 83 years old and they were tested for no major diseases (such as cancer). The age range of patients with NSCLC is between 56 and 70 years old, and they were excluded if they had the following conditions: clinically uncontrollable cardiac symptoms, other previous or concurrent malignancies, any active autoimmune disease or autoimmune disease history, previous and current history of pulmonary fibrosis, interstitial pneumonia, pneumoconiosis, radiation pneumonia, drug-related pneumonia, severe lung function impairment, or taken antibiotics within six weeks before sample collection. The feces of healthy subjects were collected from their respective homes, while the feces of patients with NSCLC were collected from the Inner Mongolia Autonomous Region People’s Hospital (Hohhot, China). All 21 clinical feces samples were self-collected by the subjects themselves. They were instructed to use a clean plastic basin to temporarily hold feces, and then take the size of a soybean into a stool collection tube. Finally, all fresh fecal samples were stored at −80 °C for subsequent metagenomic analysis.

### 4.2. Cell Line and Culture

The LLC1 cell line was developed from the lung of a C57BL mouse that had a primary Lewis lung carcinoma tumor, and it is a commonly used cell line to develop lung cancer model (LCM) mice [47] The LLC1 cell line was purchased from Procell Life Science & Technology Co., Ltd. (Wuhan, China). The cell line was confirmed negative for *Mycoplasma* contamination. Cells were cultured in DMEM-F12 medium (Procell Life Science & Technology Co., Ltd., Wuhan, China) containing 15% fetal bovine serum (Inner Mongolia Opcel Biotechnology Co., Ltd., Hohhot, China) and 1% phosphatidylserine (Sigma-Aldrich LLC., Saint Louis, MO, USA), and grown under standard conditions of 37 °C and 5% CO_2_.

### 4.3. Mouse Model Construction

The mouse experiment and related procedures were approved by the Experimental Animal Welfare and Ethics Committee of the Inner Mongolia Agricultural University (approval number: NND20223076).

Twenty-four 6-week-old C57BL/6J female mice were purchased from Beijing HFK Bioscience Co., Ltd. (Beijing, China). All mice were maintained in a specific-pathogen-free environment in the animal facility of Inner Mongolia Agricultural University. Throughout the entire experimental period, the mice were subjected to ad-libitum feeding, and they were kept in a stable environment (temperature: 22 ± 2 °C; humidity: 50 ± 10%). All animals were acclimatized for one week before being randomly divided into two groups (*N* = 12 mice per group). The control group comprised the healthy control mice (HCM group), while the LCM group comprised lung cancer mice, with lung cancer being induced by subcutaneous injection of 5 × 10^5^ LLC1 tumor cells into the right flank of the mice. Mouse feces were collected on days 0, 7, 14, and 21 and stored at −80 °C. Starting from day 7, the tumor volume was measured every three days by measuring with a vernier caliper. The tumor volume was calculated by the formula: length × width^2^ × 0.5 [48]. On day 21, the mice were euthanized, and the spleen was collected and weighed for subsequent T cell analysis.

### 4.4. Flow Cytometry Analysis

The collected spleens were ground using a syringe piston on a 70 μm cell strainer to obtain a spleen single cell suspension, and lymphocytes were extracted using a mouse spleen lymphocyte separation kit (Thermo Fisher Scientific Inc., Shanghai, China). We extracted as much of the lymphocyte white membrane layer as possible into a new 15 mL centrifuge tube, stinted by 10 mL with PBS, and centrifuged at 300× *g*/min for 5 min. After discarding the supernatant, we added 1 mL of Roswell Park Memorial Institute (RPMI) 1640 medium (Thermo Fisher Scientific Inc., Shanghai, China) containing 10% fetal bovine serum (Inner Mongolia Opcel Biotechnology Co., Ltd., Hohhot, China) and transferred the solution to a 24-well plate. We added 2 μL of Leukocyte Activation Cocktail (BD Pharmingen, Franklin Lake, NJ, USA) and incubated the solution in the WIGGENS WCI-15R CO_2_ incubator (WIGGENS GmbH, Berlin, Germany) at 37 °C for 5 h. Afterwards, the culture solution was sucked into a 4 mL EP tube, followed by an additional 1 mL of PBS for rinsing, and was centrifuged at 300× *g*/min for 5 min. One milliliter of PBS plus 1 μL Fixed Viability Stay 700 was vortexed and reacted in room-temperature darkness for 15 min. Next, 2 mL of stain buffer (FBS, BD Pharmingen, Franklin Lake, NJ, USA) was added, centrifuged at 300× *g*/min for 5 min to remove the supernatant, and resuspended with 100 μL of FBS. In order to block non-specific antibodies, 2 μL Purified Rat Anti-Mouse CD16/CD32 was added and kept away from light at 4 °C for 7 min. Afterwards, we added corresponding volumes of surface antibodies APC-Cy7 CD3e, BV421 CD4, BV510 CD8a, and BB700 CD25 according to the recommended amount in the instruction manual, and stained at 4 °C in the dark for 30 min. We added in 2 mL of FBS to terminate the staining process. After we centrifuged and discarded the supernatant, we added 1 mL 1 × Fix/Perm Buffer to damage the nuclear membrane at 4 °C for 50 min. Then, we added 2 mL of 1 × Perm/wash buffer and centrifuged at 4 °C at 350× *g*/min for 5 min. We abandoned the supernatant, and 100 μL 1 × Perm/wash buffer and a corresponding volume of intracellular antibody FITC IFN- γ, PE-Cy7 IL-4, PE IL-17A, and APC FOXP3 were added and reacted in a dark environment at 4 °C for 45 min. Afterwards, we added 3 mL of 1 × Perm/wash buffer and retained precipitation after centrifugation. We then resuspended precipitation with 500 μL of FBS and filtered it into a 2 mL EP tube on a 70 μm cell strainer. Blank control without any antibodies was added. Flow cytometry analysis was performed on a MoFlo Astrios^EQ^ flow cytometer (Beckman Coulter Inc., Brea, CA, USA). Detailed information on all reagents and antibodies used in this section can be found in Table S1.

### 4.5. Shotgun Metagenomics and Bioinformatics Analysis

A total of 21 human fecal samples and 96 mouse fecal samples were subjected to shotgun sequencing. Metagenomic DNA from these samples was extracted with QIAamp DNA Stool Mini Kit (QIAGEN, Hilden, Germany). The NEBNext Ultra DNA library preparation kit (New England Biolabs, Inc., Ipswich, MA, USA) was used to construct the sequencing libraries. Paired-end sequencing of the DNA libraries was performed on the Illumina HiSeq 2500 platform at Novogene Co., Ltd. (Beijing, China).

Low-quality (sequences shorter than 60 nucleotides) and host-contaminated reads were filtered out by the KneadData quality control pipeline (http://huttenhower.sph.harvard.edu/kneaddata, accessed on 4 January 2024). High-quality reads from each sample were assembled using MEGAHIT to obtain contigs [49]. Then, the MetaBAT2, VAMB, and DAS tools were used for binning contigs larger than 2000 bp [50,51]. Biners obtained from different software were merged to obtain the metagenome-assembled genomes (MAGs), and the integrity and contamination level of the MAGs were evaluated through CheckM (https://github.com/Ecogenomics/CheckM, accessed on 4 January 2024). Afterward, we clustered the MAGs to obtain species-level genomic bins (SGBs), calculated and expressed the average content of SGBs in each contig in reads per kilobase million, and annotated the SGBs using the Genome Taxonomy Database (https://gtdb.ecogenomic.org/, accessed on 4 January 2024). Metagenomic functions (i.e., relevant key gut metabolism modules, GMMs) were characterized and annotated according to published references, the MetaCyc metabolic database, and the Kyoto Encyclopedia of Genes and Genomes Orthologues database [52,53,54,55].

### 4.6. Statistical Analysis

The R software (v.4.0.2) was used for statistical evaluation. The normalized reads per kilobase million abundance table, species α diversity, non-metric multidimensional scaling (NMDS, Bray–Curtis distance), and principal coordinates analysis (PCoA, Bray-Curtis distance) were calculated using R packages vegan (v.2.5-1) and amplicon (v1.19.0). The ggpubr (v0.6.0) package was used to evaluate changes in the microbial community structure. Other R packages, including reshape2 (v1.4.4), knitr (v1.42), and ggplot2 (v3.4.2), were used to draw species stacking maps. All graphical presentations were generated in the R software (v.4.0.2) and Adobe Illustrator 2020 environments.

## Figures and Tables

**Table 1:** Significant differential gut metabolic modules (GMMs) between the fecal microbiota of subjects with non-small cell lung cancer (NSCLC) and healthy control (HC).

Pathway		mean_NSCLC (Arbitrary Units)	mean_HC (Arbitrary Units)	sd_NSCLC (Arbitrary Units)	sd_HC (Arbitrary Units)	Benjamini-Hochberg Corrected *P*, Wilcoxon Test
GMM010	Butyrate synthesis I	53,993,846	113,000,000	31,444,828	9,977,743	0.000316
GMM033	Melatonin synthesis	24,724,187	57,855,027	15,305,095	5,420,989	0.000316
GMM013	Isovaleric acid synthesis II (KADC pathway)	50,297,742	120,000,000	29,493,292	12,180,467	0.000316
GMM038	GABA synthesis III	22,555,066	55,021,164	12,773,931	6,999,335	0.000316
GMM039	GABA degradation	35,005,566	73,710,252	19,381,261	4,117,453	0.000316
GMM030	Quinolinic acid degradation	182,000,000	394,000,000	101,000,000	29,381,499	0.000316
GMM018	PUFAs synthesis (AA, EPA, DHA)	99,607,290	229,000,000	56,642,838	20,936,594	0.000316
GMM021	Tryptophan synthesis	8,385,742	18,034,299	4,697,625	2,370,340	0.000316
GMM001	Acetate synthesis I	220,000,000	475,000,000	123,000,000	36,742,125	0.000316
GMM005	Acetate degradation	58,489,189	131,000,000	32,551,505	14,959,711	0.000316
GMM008	Propionate synthesis III	15,614,304	35,211,328	9,076,876	4,091,735	0.000316
GMM014	Secondary bile acid biosynthesis	3,438,786	9,772,027	2,274,418	1,859,919	0.000357
GMM007	Propionate synthesis II	19,529,539	42,233,217	11,149,791	5,828,594	0.000357
GMM022	Tryptophan degradation	14,821,305	31,203,584	9,182,576	5,132,620	0.00071
GMM003	Acetate synthesis III	6,374,973	15,170,133	4,088,844	3,289,936	0.00071
GMM012	Isovaleric acid synthesis I (KADH pathway)	1,526,150	5,838,374	1,354,434	3,053,645	0.000819
GMM040	Menaquinone synthesis (vitamin K2) I	29,799,019	57,445,013	18,151,028	6,611,267	0.000819
GMM041	Menaquinone synthesis (vitamin K2) II	3,088,360	7,473,864	2,634,995	2,105,237	0.001303
GMM002	Acetate synthesis II	185,558.3	665,099.1	628,913	951,112.1	0.002039
GMM011	Butyrate synthesis II	246,549.1	1,263,851	578,002.3	1,082,580	0.002473
GMM015	Secondary bile acid biosynthesis	3,571,747	6,600,120	2,455,394	1,036,932	0.003611
GMM019	Histamine synthesis	7,938,246	12,843,787	4,291,302	1,504,558	0.003611
GMM020	Histamine degradation	923,024	2,241,686	1,186,521	1,610,406	0.040182

## Data Availability

Data is contained within the article and Supplementary Material.

## Supplementary Materials

The following supporting information can be downloaded at: https://www.mdpi.com/article/10.3390/ijms25073768/s1.

## Abbreviations

NSCLC (Non-small cell lung cancer); HC (Healthy control); LLC (Lewis lung carcinoma); LCM (Lung cancer model); HCM (Healthy control mice); PBS (Phosphate buffer saline); FBS (Fetal bovine serum); MAG (Metagenome-assembled genome); SGB (Species-level genomic bin); GMM (Gut metabase module); NMDS (Non-metric multidimensional scaling); PCoA (Principal co-ordinates analysis); SCFA (short chain fatty acid).
