# Supplementary figures and images for: Dynamic Changes of the Gut Microbiota and Its Functional Metagenomic Potential during the Development of Non-Small Cell Lung Cancer
Source: Int J Mol Sci. 2024 Mar 28;25(7):3768. doi: 10.3390/ijms25073768 (PMC11011768; doi:10.3390/ijms25073768)

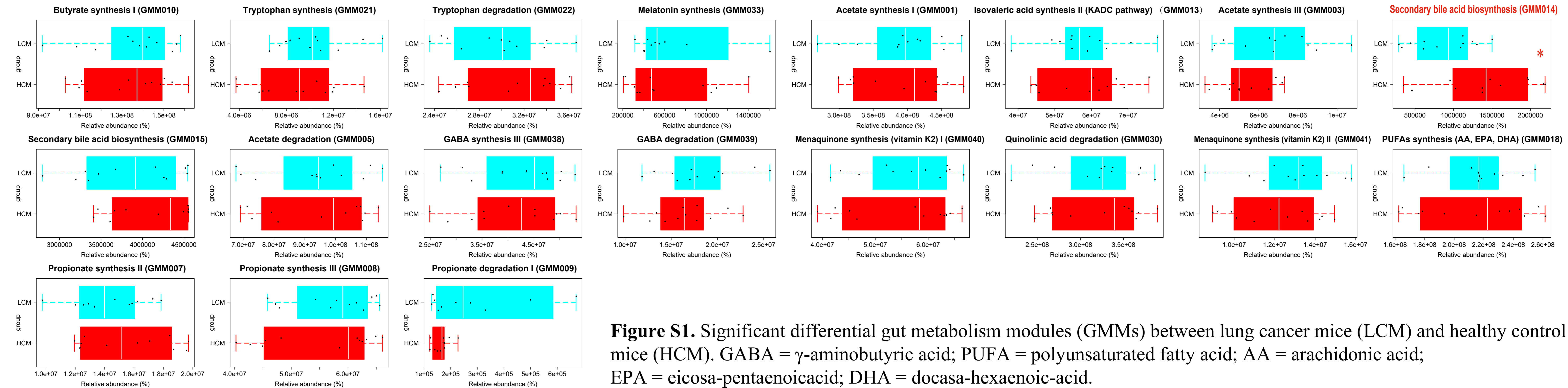

Supplement: Supplementary file 1 [file ijms-25-03768-s001.zip › Figure S1.pdf]
